# Care of Teeth of Children

**Published:** 1897-06

**Authors:** 


### CARE OF TEETH OF CHILDREN.

Begin early with the deciduous teeth and aim to keep them constantly under care. The parents should be instructed to send their children to the dentist at least once every six months. Except in rare instances, deciduous teeth should not be extracted until absorption has been completed and nature has practically thrown them off, or, failing to do so, demands assistance; it is well known that in the alveolar ridge, as in a stone arch, the removal of one part allows the rest to fall in, and that when a tooth has been prematurely extracted there is less room for the eruption of the permanent one; the incoming tooth not only has to overcome this difficulty, but it is also deprived of the nourishment it should receive in the decalcification going on about it. The deciduous teeth are not very sensitive until about the fourth year, when decalcification begins, after which the roots have jagged, sharp points, which may be a source of trouble. Mothers should be advised of these facts so that they may restrain their children from crushing hard substances in the mouth. Nuts and cheap candies are very injurious in this respect.—*Dental Digest in Medical Age.*

